# Exploring facilitators and barriers to self-management engagement of Chinese people with type 2 diabetes mellitus and poor blood glucose control: a descriptive qualitative study

**DOI:** 10.1186/s12902-022-01214-0

**Published:** 2022-11-26

**Authors:** Yuan Liu, Jiajia Jiang, Wenjun You, Dandan Gong, Xiaoqing Ma, Min Wu, Feng Li

**Affiliations:** 1 National Metabolic Management Center, Institute for Chronic Disease Management, Jining No. 1 People’s Hospital, Jining, China; 2 Department of Endocrinology, Jining No. 1 People’s Hospital, Jining, China

**Keywords:** Type 2 diabetes, Self-management, Qualitative study

## Abstract

**Aims:**

To explore facilitators and barriers to self-management engagement of Chinese people with poorly controlled type 2 diabetes.

**Methods:**

Purposive sampling method was used for recruitment. Semi-structured interview and thematic analysis was used for data collection and analysis.

**Results:**

Twenty-six semi-structured interviews were conducted. Poor blood glucose control introduced awareness of susceptibility to complications, while mental disorders could be concomitant. General knowledge about healthy lifestyle and unhealthy habits impeded lifestyle management. Temporary remission of hyperglycemia and no perceived symptoms interfered engagement of medication therapy and regular blood glucose monitoring. Family and work environments could impact self-management engagement. Accessibility to reliable diabetes-related information influenced self-management engagement.

**Conclusions:**

Awareness of susceptibility to complications motivated self-management engagement, while the awareness could cause mental disorders that need to be addressed.

Customized lifestyle plans and behavior change technologies were crucial for lifestyle management. The progression of diabetes, importance of continuity of medication therapy, and the value of blood glucose monitoring should be clarified in diabetes education.

Building diabetes-friendly social environments and providing reliable diabetes-related information were essential.

**Supplementary Information:**

The online version contains supplementary material available at 10.1186/s12902-022-01214-0.

## Introduction

Diabetes is a public health challenge leading to enormous burdens on global health care systems [1]. The latest national survey reported that 1.7 billion Chinese adults suffered from diabetes in 2018, and approximately 90% of the diabetes was diagnosed as type 2 diabetes mellitus (T2DM) [2]. Despite the prevalence of T2DM, blood glucose control is still suboptimal in Chinese people with T2DM [3, 4]. The optimal target of blood glucose control of Chinese people with T2DM was defined as HbA1c < 53 mmol/mol (7%) [2]. However, 77% of Chinese people with T2DM had poor blood glucose control (HbA1c > 7%) [3–5]. Because poor blood glucose control could cause severe complications and premature death, improving blood glucose control is worthy of more attention [6].

Self-management has been proven as the cornerstone to improve blood glucose control [7–14]. Self-management refers abilities of people with chronic diseases to collaborate with health care teams and social networks to cope with their health conditions [9].

However, diabetes self-management engagement is significantly inadequate in Chinese people with T2DM [11]. Merely 9.2–16.7% of Chinese people with T2DM had adequate self-management engagement [3, 4]. Given this situation, prompting self-management engagement could be important [15]. To prompt self-management engagement, exploring factors impacting self-management engagement of Chinese people with poorly controlled diabetes is essential [16–18].

The qualitative studies that explored factors impacting self-management engagement of people with diabetes have been conducted in the United States, European countries, the UK, Latin America, Africa, and South Asia [19–29]. These studies found that patients’ perceptions of diabetes diagnosis, mental disorders, knowledge about diabetes and self-care behaviors, and social support could impact self-management.

Patients’ perceptions of diabetes diagnosis could determine the motivation of self-management engagement [19, 21, 22, 24, 27]. The patients’ perceptions of diabetes diagnosis were defined as patients’ attitudes to the severity of being diagnosed as diabetes and intentions to taking actions to cope with the diagnosis [19, 21, 22, 24, 27]. People who perceived the diagnosis as a severe condition could be motivated to engage in health-related behaviors to cope with diabetes [19, 21, 22, 24, 27]. However, perception of diabetes diagnosis could be culturally sensitive [19, 22, 24, 27]. Diabetes diagnosis could be regarded as a natural process of life and a mild condition [19, 22, 24, 27]. This perception caused negative attitude toward health-related behaviors [19, 22, 24, 27]. Although the influences of perceptions of diabetes diagnosis were recognized, patients’ perceptions of poor blood glucose status and how the status influences self-management engagement are still unclear.

Mental disorders impacted self-management engagement [19, 22]. The mental disorders was defined as mental health issues that were prevalent in people with diabetes [19, 22].. The mental disorders included depression, anxiety, and diabetes distress [19, 22]. The mental disorders have been proven to reduce self-management engagement [19, 22]. However, the correlation between mental disorders and poor blood glucose control needs more exploration.

Providing patients with knowledge about diabetes and self-care behaviors could increase the awareness of necessity of self-management, which motivated self-management engagement [19–29]. However, people with diabetes could be reluctant to obtain new knowledge about diabetes and to use the knowledge to manage own diabetes [21, 25, 28]. This might be caused by that the knowledge was inconsistent with personal demands and was not culturally sensitive [19, 21–29]. Given this situation, it is necessary to identify the knowledge demands of population with specific contextual characteristics, while the demands of Chinese people with poorly controlled diabetes need further exploration.

Social support could increase self-management engagement [19–27, 29]. However, the demands of social support on diabetes self-management engagement were various across populations of different continents [19–22, 24–27, 29]. Social support demands on self-management engagement are still unclear in Chinese population.

In summary, the influences of poor blood glucose status and mental health are still unclear. Demands of diabetes knowledge and social support in Chinese population need further exploration.

To fulfill the research gap, this qualitative study aimed to explore facilitators and barriers to self-management engagement of Chinese people with T2DM and poor blood glucose control. It was anticipated the findings of this study would provide in-depth understanding to self-management engagement of Chinese people with poorly controlled T2DM, which could be useful to develop customized interventions to improve self-management engagement and blood glucose control.

## Materials and methods

### Research design

This study was designed according to qualitative descriptive research methods. Qualitative descriptive methods are typically amenable to providing a comprehensive understanding of unknown phenomena, which are relevant to healthcare practice and health policies, such as factors impacting health behaviors and health services usage [30, 31]. Consequently, qualitative descriptive design is consistent with research aim of this study. According to the guidance of qualitative descriptive study design, purposive sampling method was used for participant recruitment [32, 33]. Specifically, maximum variation sampling was used to obtain broad and rich information of interest [32, 33]. Semi-structured interview method was used to collect qualitative data [32, 33]. Thematic analysis was used to analyze the data collected [31, 32, 34].

### Participants and recruitment

**Fig. 1:**
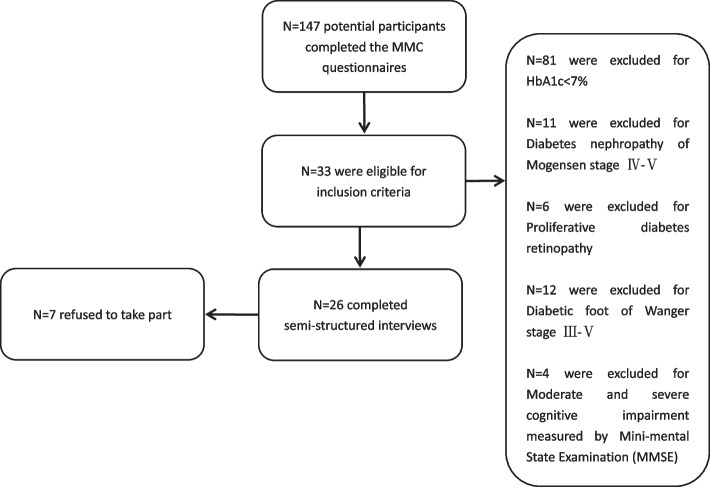
Recruitment Process

Purposive sampling method was used to recruit participants who could provide information of interest and to achieve maximum demographic variations of the participants [35, 36]. Eligible participants were recruited from the patients who were referred to outpatient clinics of the National Metabolic Management Center (MMC). MMC is an independent medical care institution providing services for patients who are referred from both primary and second care settings. The patients referred to MMC were contacted in person at the outpatient clinics of MMC by the first author. Demographic and biochemical information of the patients was collected. According to the information, purposive sampling was conducted to select participants who could provide knowledge of interest and achieve demographic variation maximum. The recruitment process was assisted by endocrinologists and diabetes specialist nurses who were working in the outpatient clinics of MMC. The recruitment process was available in Fig. 1. Recruitment process.

The explicit inclusion/exclusion criteria were described in Table 1 Inclusion/exclusion criteria. The sample size depends on the stage where data saturation was reached, which means newly recruited participants cannot provide new information and knowledge relating to the study topic [37].

**Table 1:** Inclusion/exclusion criteria

Inclusion criteria	Exclusion criteria
People over 18 years old	Diabetes nephropathy of Mogensen stage IV-V
People diagnosed with type 2 diabetes more than 1 year	Proliferative diabetes retinopathy
People with poor blood glucose control (HbA1c > 7%)	Diabetic foot of Wanger stage β-V
	Coronary heart disease of NYHA stage β-IV
	Moderate and severe cognitive impairment measured by Mini-mental State Examination (MMSE)

### Theoretical framework

Health Belief Model (HBM) was used as the theoretical foundation and framework to guide interviews and data analysis in this study. The HBM is a social-psychological model that was commonly used in health research [38]. It is fundamentally a ‘value-expectancy’ model developed to understand health-related behavior of individuals under specific health conditions [38]. The HBM advocated that perceived value of health improvement and expectancy that specific health-related behavior would ameliorate health conditions would determine health behavior engagement [38]. According to the HBM, health-related behavior engagement is determined by four dimensions [38]: perceived susceptibility, perceived severity, perceived benefits, and perceived barriers [38].

**Fig. 2:**
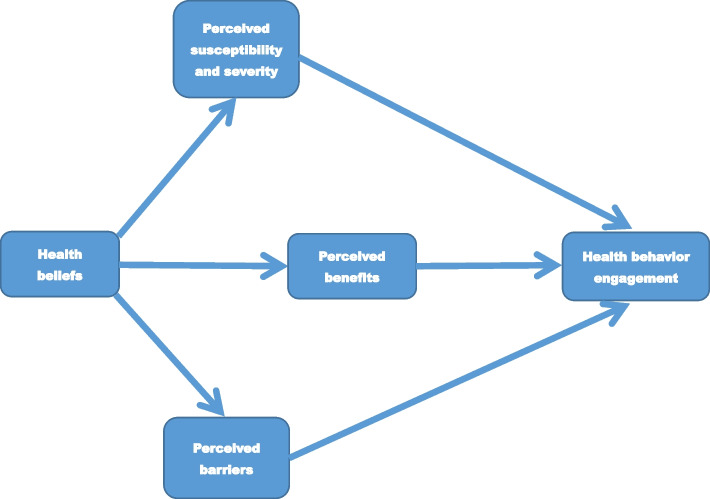
Health Belief Model

Perceived susceptibility and severity refer to individuals’ beliefs about personal risk to develop specific illnesses and subjective perceptions of seriousness of the illnesses respectively [38]. Perceived benefits were defined as people’s perceptions about benefits of specific health-related behaviors or treatment regimens on improving their health conditions [38]. Perceived barriers mean practical hindrances that could obstacle health behavior uptake and engagement [38]. The framework of HBM is accessible in Fig. 2. Health Belief Model. Interview outline and data analysis were framed by HBM.

### Data collection

Twenty-six participants were recruited and 26 semi-structured interviews were conducted. The first and second authors independently conducted the interviews. The pre-defined interview outline guided the interviews. The interview outline was formulated by the research group according to the HBM framework. The original interview outline was iteratively revised through discussions among members of the research group that included endocrinologists, diabetes nurses, diabetes educators and two academic researchers. The outline was finalized when consensus was reached. The interviews were conducted at the outpatient clinics of MMC between October 2021 and January 2022. The interviews lasted approximately 30–50 minutes. The interviews were digitally recorded and were verbatim transcribed. The interview outline is available in Appendix 1. Interview guideline.

### Data analysis

Thematic analysis method was used to analyze the qualitative data [39, 40]. The data analysis process was assisted by NVivo 12. Nvivo.12 is a powerful qualitative data analysis software. It can efficiently organize and analyze the information collected by interviews, focus groups, questionnaires, and audio. The information content include words, pictures, audio recordings, and videos.

In the first phase, the researchers read the data repetitively to become familiar with the data and generate initial ideas of what is in the data. In the second phase, initial codes of the data were generated. This phase is a ‘theory-driven’ process that the data was coded with the indication of HMB framework. In the third phase, the initial codes were sorted into potential themes according to meaning of the codes. This process was also instructed by the HBM framework. In the fourth phase, the potential themes were reviewed to check coherence of the data within the same themes and to verify whether the themes could reflect accurate meaning of the data. In the fifth phase, name of the themes was refined to ensure the name could capture essential meaning of the data sets.

Two academic researchers independently conducted the data analysis. Discrepancies between the two researchers were solved by discussions within the research group. Consensus was reached on the final themes.

### Rigor

Rigor of this study was considered from four dimensions: credibility, transferability, dependability, and confirmability [41]. Member checks were performed to enhance the credibility of study findings. Participants were engaged to review whether the themes and interpretations of the data collected were consistent with the accurate meaning of participants. Study settings and demographic characteristics of participants are available in this study to clarify the transferability of study findings. To enhance dependability of data interpretation, two academic researchers independently analyzed the data collected, and discrepancies between the two researchers were solved by research group discussions. Concerning confirmability, audit trail was maintained to record the research process. Peer audit was performed. Two senior researchers who did not involve in this study examined the research process.

### Ethics statement

This study was approved by the Committee on Human Research of the Jining NO.1 People’s Hospital (2020 Ethical Approval No. 057). All participants gave written informed consent in accordance with the Declaration of Helsinki.

## Results

Demographic characteristics of the participants are available in Table 2. Characteristics of participants. Five themes were identified: (1) Susceptibility to severe complications motivates action engagement; (2) Self-management is beneficial; (3) Barriers to self-management engagement; (4) Two sides of social environment; (5) Obtaining reliable information. The summary of the themes is available in Table 3. Summary of themes.

**Table 2:** Characteristics of participants

Demographic characteristics	Male (*n* = 15)	Female (*n* = 11)	Total (*n* = 26)
Age (mean)	44	48	46
Married	10	9	19
Primary school graduation	5	4	9
Middle school graduation	7	5	12
College university graduation	3	2	5
Employed	11	4	15
Unemployed	4	8	12
HbA1c (mean/mmol/L)	8.67	8.69	8.68
Duration of T2DM(mean/month)	72	53	64

**Table 3:** Summary of major themes

Major themes	Brief description of the themes
Susceptibility to severe complications motivates action engagement	Participants were aware of own susceptibility to severe complications that could be caused by poor blood glucose. The awareness could motivate action engagement.
Self-management is beneficial	Self-management, which included lifestyle management, medication therapy, and blood glucose monitoring, was believed by participants to be beneficial for improving blood glucose control. The perceived benefit facilitated self-management engagement.
Barriers to self-management engagement	Practical barriers to self-management engagement were identified. The barriers included: (1) Uselessness of general knowledge about lifestyle management; (2) Difficulty in changing unhealthy habits; (3) Temporary remission and discontinuity of medication therapy engagement; (4) Blood glucose monitoring and physical symptoms.
Two sides of social environment	The influence of social environment on self-management engagement could be both positive and negative
Obtaining reliable information	Providing participants with reliable information about diabetes could improve self-management engagement, while reliable information was not always available

### Susceptibility to severe complications motivates action engagement

Participants’ awareness of their own susceptibility to severe complications that might be caused by poor blood glucose control could facilitate self-management engagement. All participants were aware that their poor blood glucose control status had caused susceptibility to diabetic complications. The diabetic complications were regarded as severe conditions that could impact normal functions of their bodies, such as the functions of eyes, renal, and foot.

“Now I clearly understand that my status (poor blood glucose control) is easy to develop complications. I know it would be really bad for eye and feet.” (P-16).

The concern of susceptibility to the severe complications generated participants’ intention to prevent the complications. To avoid the complications, motivation to engage in specific actions was identified.

“Yes, I just want to try my best to control it (blood glucose). I’m still young and I have to keep away from them (complications).”(P-8).

“I understand complications are very bad, I planed to take some measures to control my blood glucose, and to avoid the complications.” (P-14).

Mental health burdens were identified in some participants. The concern about poor blood glucose control and susceptibility to complications caused the Mental health burdens.

“I didn’t care before. Now I just feel that my body is not at a good status. My blood glucose was not well controlled. I’m afraid of renal complications, and other complications. I feel anxious.” (P-3).

“But my current situation (poor blood glucose control) is not good, it is also difficult to deal with it. I’m very anxious.”(P-11).

### Self-management is beneficial

The participants thought self-management was beneficial approach to improve blood glucose control. The perceived benefit motivated self-management engagement. The specific contents of self-management included: (1) Lifestyle management; (2) Medication therapy; (3) Blood glucose monitoring.

#### Lifestyle management

Motivation to engage in lifestyle management was expressed by the participants, which was triggered by the perceived benefit of lifestyle management in improving blood glucose control. Lifestyle management included eating a healthy diet and doing more exercise. The participants believed that engaging in lifestyle management was beneficial in improving blood glucose control. Some participants stated that they used to achieve good blood glucose control when they engaged in lifestyle management. The perceived benefits generated participants’ intention to engage in lifestyle management to improve blood glucose control.

“I would pay more attention to my diet. I plan to increase my intake of vegetables. I hope I could control it (blood glucose).”(P-9).

“I would try to do more exercise.”(P-15).

#### Medication therapy

The participants believed that medication therapy was the most effective approach for blood glucose control. The participants thought it was impossible to get blood glucose under control without taking medications. This perception generated a positive attitude toward medication therapy engagement.

“I used to take the medications three times a day, I could stick to the regimens at that time. If you can’t stick to the regimens, your blood sugar will be high. If you can do it, your blood sugar will be normal.”(P-7).

#### Blood glucose monitoring

Some participants showed good blood glucose monitoring engagement, which was associated with awareness of benefits of blood glucose monitoring. These participants stated that they knew blood glucose monitoring could indicate how their blood glucose was controlled and whether their diet and medication regimens demanded adjustment. Because the awareness of benefits of blood glucose monitoring, the participants showed good blood glucose monitoring engagement.

“I also knew that. I would measure my blood glucose. If it was not high, I would know how much to eat. For example, eating a boiled egg, a cake and a cup of soybean milk in the morning could ensure that the blood glucose after meals is not high.” (P-21).

“I would measure my blood glucose, to adjust my medication and diets.” (P-22).

#### Barriers to self-management engagement

Although the motivation for action engagement and belief on benefits of self-management were identified, practical barriers to self-management engagement were recognized. The barriers included four dimensions: (1) Uselessness of general knowledge about lifestyle management; (2) Difficulty in changing unhealthy habits; (3) Temporary remission and discontinuity of medication therapy engagement; (4) Blood glucose monitoring and physical symptoms.

#### Uselessness of general knowledge about lifestyle management

Providing participants with general knowledge about lifestyle management was useless for achieving successful lifestyle management engagement. Although the participants possessed general knowledge about lifestyle management, various everyday living experiences and living patterns of individual participants caused difficulty in using the general knowledge to formulate personal and practical lifestyle management schemes, which impeded lifestyle management engagement.

“I know I need to control my diets, to control energy intake and do more exercise. But all these are too general, it didn’t make sense for me because I live a life in my own way. I believed everyone has their own ways for living. I still don’t know how to make my life more health. I still don’t have my own plans for adjusting my diets and exercise.” (P-17).

#### Difficulty in changing unhealthy habits

To live the lifestyles proposed for people with diabetes, some participants stated that they had to change their unhealthy habits formed by long-term periods. Although the unhealthy habits caused poor blood glucose control, it was still a challenge to break the habits.

“My poor blood glucose control might be caused by my diets. I know what kind of food is good for me, but I just couldn’t control myself. I know I couldn’t eat too much fried food, but I like fried hairtail very much and often ate too much. After eating, you regretted it. When you ate, you just couldn’t stop it.” (P-8).

“I do little exercise in daily life, that is a habit formed for a long time.. I don’t like running, I don’t like sports, and I don’t like dancing.” (P-15).

#### Temporary remission and discontinuity of medication therapy engagement

Discontinuity of medication therapy engagement was identified. The discontinuity was caused by temporary remission of hyperglycemia and physical symptoms. Some participants stopped taking medications when their blood glucose was temporarily under control or no physical symptoms were perceived. The temporary remission was considered the signal of stopping medication therapy.

“I used to take medicine according to the regimens proposed by my doctor. I insisted on taking drugs, and my blood sugar was well controlled at that time. After that, I didn’t insist on taking medication. I felt that my condition was under control and felt no discomfort. I didn’t take medication after that.” (P-22).

Two participants stated that they did not take medication because potential side effects and drug dependence. However, the concerns did not influence medication therapy engagement of the majority of participants. Despite potential side effects and drug dependence, other participants thought taking medication to control blood glucose was more critical.

“I don’t care about side effect. I know that drugs could damage the liver and kidney, but I must control my blood glucose. I often watched videos introducing western medicine would have side effects on my mobile phone, but you couldn’t make it (good blood glucose control) without taking the medications.”(P-7).

“If you don’t take medication, it would be troublesome in case of poor blood glucose control. Now I don’t care about the side effects and dependence of drugs, because now my live is threatened and I must insist on taking drugs. I’m more worried about the poor control of blood glucose.”(P-10).

#### Blood glucose monitoring and physical symptoms

The majority of participants in this study did not engage in regular blood glucose monitoring because the function of blood glucose monitoring was misunderstood. The participants believed that blood glucose monitoring should only be performed when physical symptoms occurred. The participants thought it was unnecessary to measure their blood glucose when no physical symptoms were perceived. No perceived physical symptoms was considered as an indicator of healthy status. Because the healthy status, blood glucose monitoring was believed to be unnecessary. The participants would only measure blood glucose when physical symptoms were perceived.

“I didn’t feel uncomfortable before, but now I feel it. I can’t see clearly, and my feet are swollen. It was only when the body began to change that I began to do it (blood glucose monitoring).” (P-19).

“Well, I only measured my blood glucose when I felt uncomfortable. I didn’t measure it when I felt good. I didn’t measure it regularly.” (P-24).

### Two sides of social environment

The social environment of participants was identified as both facilitator and barrier to self-management engagement. The social environment included family and work environment.

The roles of family environment were determined by how family members influenced self-management. On one side, the family members who had positive attitudes toward assisting the participants in managing diabetes could facilitate self-management engagement. These family members facilitated self-management engagement by playing the role of offering healthy diets, promoting physical activity engagement, and reminding blood glucose monitoring.

“I’m living with my wife. My wife made a healthy lifestyle plans for me. She wanted me to do more exercise, to eat more vegetables. And I would try to do what she wants me to do.” (P-11).

“My family was worried about my current health status. They wanted me to have a healthy diet. For me to have a healthy diet, my daughter formulated a diabetes diet plans for me.” (P-18).

“My family would remind me to measure my blood sugar regularly and do more exercise.” (P-27).

On the other side, unhealthy living habits of family members could reduce self-management engagement. The unhealthy living habits could lead to a diabetes-unfriendly family environment that could impede self-management.

“My family’s eating habits are not healthy, but I have to live in the way as my family live because we are living together now. My family doesn’t understand what kinds of diets were good for diabetes, they can give me no support.” (P-13).

“There are no people around who love sports. My family doesn’t like sports either. No one urged me to exercise. My family doesn’t exercise either.” (P-23).

Work environment where healthy diet and exercise facilities were accessible in workplaces could facilitate self-management engagement. Some participants stated that the availability of healthy diet and exercise facilities in workplaces increased convenience of self-management engagement.

“I work in school. Our school has a gym for employees. I could do exercises during the leisure times.” (P-4).

“The diets are OK. The place where I work provide diabetes meals, and I could have diabetes meals during work times.” (P-11).

However, restrictions on accessing healthy diet and doing exercise in workplaces were also identified. Some participants expressed that the diet provided in workplaces was unsuitable for people with diabetes, which caused difficulty in engaging in lifestyle management. Furthermore, exercise opportunities were limited because tight work schedules. These restrictions impeded self-management engagement.

“Now I work for nearly 11 hours a day. I had no time to exercise outside of work, and I were very tired after work.” (P-2).

“I didn’t eat regularly when I were on business. But I often went on business because of work. When I went on business, my diet was not easy to control, because I need to eat with my clients and my colleagues, but the food was not suitable for me.” (P-8).

Barriers to medication taking and blood glucose monitoring in workplaces were identified.

“However, it was inconvenient at work because the equipment was not easy to carry with you.” (P-12).

“I have the problem of insulin injections, because I work in sale department, sometimes insulin couldn’t be injected on time, and it was also a problem to bring it to workplace.” (P-19).

### Obtaining reliable information

Obtaining diabetes-related information could facilitate self-management engagement. The information included diabetic complications, lifestyle advice, and effects of medications. This information clarified the necessity of diabetes self-management and how diabetes could be managed, which might facilitate self-management engagement.

However, some information obtained from various resources was unreliable, especially information from the internet and social networks. Some participants stated that their friends and colleagues told them diet management was unnecessary for people with diabetes. Furthermore, some participants expressed that they obtained information from the internet that advertised health products could instead of medications for blood glucose control. This information could form incorrect perceptions and attitudes of participants regarding diabetes self-management, which might reduce self-management engagement. Compared with the information from the internet and social networks, participants preferred to obtain information from healthcare professionals.

“I usually look through the information on the internet, but I needed to check the correctness of the information by myself. If I could communicate directly with my doctors, there would be no such problems. After all, they are professional. It would be great if you could get information about diabetes directly from the medical staff.” (P-17).

## Discussion

With our knowledge, it is the first study that explored factors impacting self-management engagement in Chinese people with T2DM and poor blood glucose control. Five relevant themes were developed.

The participants’ concern about susceptibility to severe complications motivated self-management engagement. This finding is consistent with the principle of HBM, which demonstrated that perceived susceptibility and severity of specific diseases could motivate health behavior engagement [42]. However, this finding is different from a previous study that reported that Chinese people with diabetes tended to be blindly optimistic about their health conditions [43]. The inconsistent findings could be caused by this study focused on people with poor blood glucose control, which induced the concern about the susceptibility to severe complications [42, 44, 45].

This study also found that participants’ awareness of susceptibility to severe complications might increase the risk of mental disorders. Previous studies have demonstrated that awareness of susceptibility to diabetic complications could cause mental disorders [42–53], while mental disorders could reduce diabetes self-management engagement [15]. In terms of motivating self-management engagement, the balance between increasing awareness of susceptibility to complications and reducing risk of mental health disorders is still unclear and needs further exploration.

Benefits of self-management were perceived by the participants, which generated motivation to engage in self-management. This finding is consistent with the HBM which states that patients incline to engage in health behaviors that are perceived to be beneficial [42].

Although the perceived benefit could motivate health behavior engagement, according to the HBM, barriers impeding health behavior engagement could exist [42]. This point was also proven in this study. The barrier of using general lifestyle knowledge to achieve successful lifestyle management was recognized. This barrier might be caused by diverse contextual variations of the participants. The diverse contextual variations led to individual everyday living experiences and patterns. The personal living experiences and patterns could make general knowledge about lifestyle management useless for formulating personal and practical lifestyle management schemes [42, 44, 47–49], which caused the barrier to self-management engagement.

Concerning the barrier of changing unhealthy behaviors, changing the unhealthy behaviors was considered as the core value and critical component of diabetes self-management, while behavior change is challenging [51, 54]. Given this situation, behavior change technologies were widely used in diabetes management, and the effects of blood glucose control has been proven [43, 51, 52]. Consequently, behavior change technologies should integrate in diabetes self-management services.

Discontinuity of medication therapy engagement was mainly caused by temporary remission of hyperglycemia and physical symptoms. A previous study also reported that temporary remission of hyperglycemia and no subjective feelings could impede medication taking in Chinese people with diabetes [43]. The findings could be caused by lack of knowledge about pathophysiology and natural progression of diabetes. Lack of the knowledge might lead to the misunderstanding that temporary remission of hyperglycemia and no perceived physical symptoms indicated diabetes was cured and medication therapy could be stopped [43–45, 53].

Concern about side effects might reduce medication therapy engagement [43, 45, 53]. However, in this study, the concern did not impact medication therapy engagement of the majority of participants because they thought taking medications to improve blood glucose control was the more important thing. The inconsistent findings might be associated with the characteristics of participants. This study merely involved participants with poor blood glucose control. Because the poor blood glucose control status, the participants tended to put taking medications at a high-priority rating.

Most participants in this study showed poor engagement in regular blood glucose monitoring. The participants thought it was unnecessary to measure blood glucose when no subjective symptoms were perceived. A previous study also reported that having no severe symptoms or events could impede blood glucose monitoring of Chinese people with diabetes [43]. However, in this study, some participants showed good blood glucose monitoring engagement because they understood the value of regular blood glucose monitoring on adjusting lifestyle and medication regimens. Consequently, clarifying the value and normal function of regular blood glucose monitoring could be crucial.

Family environment was identified as both facilitator and barrier of self-management engagement, which was also proven by other previous studies [45, 54, 55]. This study found that family members’ attitudes and perceptions on diabetes management influenced self-management engagement. However, the finding was still preliminary, and further studies are required to explore depth understanding of how the interplay between Chinese people with diabetes and their family members impacts self-management engagement.

Similarly, this study also found that work environment where healthy diet and exercise facilities were available could prompt self-management engagement. At the same time, work restrictions such as time restriction could impede self-management engagement. Consequently, building diabetes-friendly work environment could be necessary [56, 57].

Participants in this study reported various resources for obtaining diabetes-related information, while some resources were unreliable. Although the reliable information is important for self-management engagement [55], inaccurate information was disseminated to participants in this study. The inaccurate information was mainly obtained from social networks. Although the information provided by healthcare professionals was valued, the information from social networks could be more accessible in daily life, while reliability of the information can not be guaranteed. Given this situation, channels for providing reliable information are necessary [45].

### Policy implications

Poor blood glucose control screening was suggested to introduce. The recent national survey reported that most Chinese people with poorly controlled diabetes were still unaware of their poor blood glucose control [2]. Although the perception of susceptibility to complications caused by own poor blood glucose control could motivate self-management engagement [22, 44, 45, 53], the low knowing rate of own poor blood glucose control status might impede self-management engagement. Consequently, poor blood glucose control screening were suggested.

Interventions to address mental disorders that are common in people with diabetes, which included depression, anxiety, and diabetes distress, was suggested to integrate into current Chinese diabetes services. This study found that poor blood glucose might cause the mental disorders, which reduced self-management engagement. Consequently, addressing the mental disorders is essential. However, the MMC and National Basic Public Health Service do not provide mental health services [58]. Given this situation, mental health services should be added.

Current diabetes lifestyle management services should be adopted a person-centered pattern. This study found that patients’ demands for knowledge and information about lifestyle management were personal. Consequently, providing person-centered diabetes lifestyle management services is important [42, 47–50, 59–62]. However, current Chinese healthcare professionals still incline to provide general and repeated materials to support lifestyle management [50, 59, 62]. Given this situation, introducing person-centered lifestyle management was suggested.

Behavior change technologies should integrate into current diabetes services. This study identified the difficulty in changing unhealthy behaviors. Behavior change technologies could be the appropriate approach to address the difficulty. However, behavior change technologies are rarely used in current Chinese diabetes services [43, 52, 59, 62]. Consequently, behavior change technologies should be added.

The participants showed poor knowledge about the natural progressions of diabetes and the value of regular blood glucose monitoring, which reduced self-management engagement. As a result, Chinese diabetes education services should pay more attention to the knowledge deficits.

This study identified the influences of family and work environment on self-management engagement. The influences indicated the importance of building a diabetes-friendly family and work environment [56, 57, 63–66]. Building a channel that disseminates authoritative and reliable diabetes-related is also crucial [44, 67, 68].

## Limitations

This study has some limitations. Selection bias might exist in this study. The recruitment process reflected that some patients were willing to participate in this study while others refused to participate in this study. The experiences and perspectives of the participants could be different from those who refused to participate.

Transferability of the study findings could be limited in some degree. The demographic and clinical characteristics, as well as cultural background of participants in this study should be taken into account when transferring the findings into other population.

This study merely recruited diabetic people with poor blood glucose control. Further exploration to compare people with poor and good blood glucose control was required. Furthermore, the average duration of diabetes of participants in this study was 64 months, which indicated people with newly diagnosed and short-term diabetes could be under representative. Consequently, future studies targeting on people with newly diagnosed and short-term diabetes are required.

This study explored overall self-management behaviors. The findings of specific components of the behaviors, such as exercise and diet, could be preliminary. Future studies are needed to obtain in-depth understanding to the specific components of self-management.

## Conclusion

Increasing awareness of own poor blood glucose status and susceptibility to complications is a promising strategy to motivate self-management engagement of Chinese people with poorly controlled T2DM. The accompanying mental disorders should be noticed.

To achieve successful lifestyle management engagement, formulating customized lifestyle management plans was suggested. Furthermore, behavior change technologies were suggested to integrate into existing Chinese diabetes services.

Chinese diabetes education services should pay more attention to the natural progression of diabetes and the importance of medication therapy continuity. Furthermore, the value of blood glucose monitoring should be clarified.

Policies for building a diabetes-friendly family and work environment should be considered. Building a channel that provides reliable diabetes-related information and is accessible in routine life is also important.

## Supplementary Information

**Additional file 1.** The guideline listed open-ended questions asked in the semi-structured interviews.

## Data Availability

The data that support the findings of this study are available on reasonable request from the corresponding author. The data are not publicly available due to their containing information that could compromise the privacy of research participants.

## Abbreviations

T2DM: type 2 diabetes mellitus
HBM: Health Belief Model
HbA1c: Glycosylated hemoglobin
MMC: National Metabolic Management Center

## References

[1] Chai S, Yao B, Xu L, Wang D, Sun J, Yuan N, Zhang X, Ji L (2018). The effect of diabetes self-management education on psychological status and blood glucose in newly diagnosed patients with diabetes type 2. Patient Educ Couns.

[2] Wang L, Peng W, Zhao Z, Zhang M, Shi Z, Song Z, Zhang X, Li C, Huang Z, Sun X, Wang L, Zhou M, Wu J, Wang Y (2021). Prevalence and treatment of diabetes in China, 2013-2018. JAMA.

[3] Lin K, Park C, Li M, Wang X, Li X, Li W, Quinn L (2017). Effects of depression, diabetes distress, diabetes self-efficacy, and diabetes self-management on glycemic control among Chinese population with type 2 diabetes mellitus. Diabetes Res Clin Pract.

[4] Ji M, Ren D, Gary-Webb TL, Dunbar-Jacob J, Erlen JA (2019). Characterizing a sample of Chinese patients with type 2 diabetes and selected health outcomes. Diabetes Educ.

[5] Cheng L, Sit JWH, Choi KC, Chair SY, Li X, Wu Y, Long J, Tao M (2018). Effectiveness of a patient-centred, empowerment-based intervention programme among patients with poorly controlled type 2 diabetes: a randomised controlled trial. Int J Nurs Stud.

[6] Global report on diabetes. (2021). Retrieved 23 December 2021, from https://www.who.int/publications-detail-redirect/9789241565257

[7] Gagliardino JJ, Aschner P, Baik SH, Chan J, Chantelot JM, Ilkova H, Ramachandran A, IDMPS investigators (2012). Patients' education, and its impact on care outcomes, resource consumption and working conditions: data from the international diabetes management practices study (IDMPS). Diabetes Metab.

[8] Gagliardino JJ, Lapertosa S, Pfirter G, Villagra M, Caporale JE, Gonzalez CD, Elgart J, González L, Cernadas C, Rucci E, Clark C, Jr; PRODIACOR. (2013). Clinical, metabolic and psychological outcomes and treatment costs of a prospective randomized trial based on different educational strategies to improve diabetes care (PRODIACOR). Diabet Med.

[9] Richard AA, Shea K (2011). Delineation of self-care and associated concepts. J Nurs Scholarsh.

[10] Minet L, Møller S, Vach W, Wagner L, Henriksen JE (2010). Mediating the effect of self-care management intervention in type 2 diabetes: a meta-analysis of 47 randomised controlled trials. Patient Educ Couns.

[11] Guo XH, Yuan L, Lou QQ, Shen L, Sun ZL, Zhao F, Dai X, Huang J, Yang HY (2012). Chinese diabetes education status survey study group. A nationwide survey of diabetes education, self-management and glycemic control in patients with type 2 diabetes in China. Chin Med J.

[12] Zhang XX, Wu SY, Wang FB, Yu SP, Sun KG, Hu K, et al. Association between social support and self-management behaviors among patients with diabetes in community. Beijing Da Xue Xue Bao. 2017;49:455–61. 28628147

[13] Povey RC, Clark-Carter D (2007). Diabetes and healthy eating: a systematic review of the literature. Diabetes Educ.

[14] Odegard PS, Capoccia K (2007). Medication taking and diabetes: a systematic review of the literature. Diabetes Educ.

[15] Luo X, Liu T, Yuan X, Ge S, Yang J, Li C, Sun W (2015). Factors influencing self-Management in Chinese Adults with type 2 diabetes: a systematic review and Meta-analysis. Int J Environ Res Public Health.

[16] DePue JD, Rosen RK, Batts-Turner M, Bereolos N, House M, Held RF, Nu'usolia O, Tuitele J, Goldstein MG, McGarvey ST (2010). Cultural translation of interventions: diabetes care in American Samoa. Am J Public Health.

[17] Feathers JT, Kieffer EC, Palmisano G, Anderson M, Janz N, Spencer MS, Guzman R, James SA (2007). The development, implementation, and process evaluation of the REACH Detroit Partnership's diabetes lifestyle intervention. Diabetes Educ.

[18] Wilson C, Alam R, Latif S, Knighting K, Williamson S, Beaver K (2012). Patient access to healthcare services and optimisation of self-management for ethnic minority populations living with diabetes: a systematic review. Health Soc Care Community.

[19] Suglo JN, Evans C (2020). Factors influencing self-management in relation to type 2 diabetes in Africa: a qualitative systematic review. PLoS One.

[20] Murphy K, Casey D, Dinneen S, Lawton J, Brown F (2011). Participants' perceptions of the factors that influence diabetes self-management following a structured education (DAFNE) programme. J Clin Nurs.

[21] Frost J, Garside R, Cooper C, Britten N (2014). A qualitative synthesis of diabetes self-management strategies for long term medical outcomes and quality of life in the UK. BMC Health Serv Res.

[22] Majeed-Ariss R, Jackson C, Knapp P, Cheater FM (2015). A systematic review of research into black and ethnic minority patients' views on self-management of type 2 diabetes. Health Expect.

[23] Schmidt SK, Hemmestad L, MacDonald CS, Langberg H, Valentiner LS (2020). Motivation and barriers to maintaining lifestyle changes in patients with type 2 diabetes after an intensive lifestyle intervention (the U-TURN trial): a longitudinal qualitative study. Int J Environ Res Public Health.

[24] Patel T, Umeh K, Poole H, Vaja I, Newson L (2021). Cultural identity conflict informs engagement with self-management Behaviours for south Asian patients living with Type-2 diabetes: a critical interpretative synthesis of qualitative research studies. Int J Environ Res Public Health.

[25] Christensen NI, Drejer S, Burns K, Lundstrøm SL, Hempler NF (2020). A qualitative exploration of facilitators and barriers for diabetes self-management behaviors among persons with type 2 diabetes from a socially disadvantaged area. Patient Prefer Adherence.

[26] Saunders T (2019). Type 2 diabetes self-management barriers in older adults: an integrative review of the qualitative literature. J Gerontol Nurs.

[27] Blasco-Blasco M, Puig-García M, Piay N, Lumbreras B, Hernández-Aguado I, Parker LA (2020). Barriers and facilitators to successful management of type 2 diabetes mellitus in Latin America and the Caribbean: a systematic review. PLoS One.

[28] Fransen MP, Beune EJ, Baim-Lance AM, Bruessing RC, Essink-Bot ML (2015). Diabetes self-management support for patients with low health literacy: perceptions of patients and providers. J Diabetes.

[29] Catapan SC, Nair U, Gray L, Cristina Marino Calvo M, Bird D, Janda M, Fatehi F, Menon A, Russell A (2021). Same goals, different challenges: a systematic review of perspectives of people with diabetes and healthcare professionals on type 2 diabetes care. Diabet Med.

[30] Colorafi KJ, Evans B (2016). Qualitative descriptive methods in health science research. HERD.

[31] Kim H, Sefcik JS, Bradway C (2017). Characteristics of qualitative descriptive studies: a systematic review. Res Nurs Health.

[32] Neergaard MA, Olesen F, Andersen RS, Sondergaard J (2009). Qualitative description - the poor cousin of health research?. BMC Med Res Methodol.

[33] Sandelowski M. Whatever happened to qualitative description? Res Nurs Health. 2000;23:334–40. https://pubmed.ncbi.nlm.nih.gov/10940958/. 10.1002/1098-240x(200008)23:4<334::aid-nur9>3.0.co;2-g 10940958

[34] Vaismoradi M, Turunen H, Bondas T (2013). Content analysis and thematic analysis: implications for conducting a qualitative descriptive study. Nurs Health Sci.

[35] Tongco M (2007). Purposive sampling as a tool for informant selection. Ethnobot Res Appl.

[36] Suen LJ, Huang HM, Lee HH (2014). A comparison of convenience sampling and purposive sampling. Hu Li Za Zhi.

[37] Wuest J (2011). Are we there yet? Positioning qualitative research differently. Qual Health Res.

[38] Becker MH, Janz NK (1985). The health belief model applied to understanding diabetes regimen compliance. Diabetes Educ.

[39] Braun V, Clarke V (2019). Reflecting on reflexive thematic analysis. Qual Res Sport Exerc Health.

[40] Clarke V, Braun V (2018). Using thematic analysis in counselling and psychotherapy research: a critical reflection. Couns Psychother Res.

[41] Moon MD, Wolf LA, Baker K, Carman MJ, Clark PR, Henderson D, Manton A, Zavotsky KE (2013). Evaluating qualitative research studies for use in the clinical setting. J Emerg Nurs.

[42] Bech LK, Borch Jacobsen C, Mathiesen AS, Thomsen T (2019). Preferring to manage by myself: a qualitative study of the perspectives of hardly reached people with type 2 diabetes on social support for diabetes management. J Clin Nurs.

[43] Taj F, Klein MCA, van Halteren A (2019). Digital health behavior change technology: bibliometric and scoping review of two decades of research. JMIR Mhealth Uhealth.

[44] Karimi Moonaghi H, Namdar Areshtanab H, Jouybari L, Arshadi Bostanabad M, McDonald H (2014). Facilitators and barriers of adaptation to diabetes: experiences of Iranian patients. J Diabetes Metab Disord.

[45] Chepulis L, Morison B, Cassim S, Norman K, Keenan R, Paul R, Lawrenson R (2021). Barriers to diabetes self-Management in a Subset of New Zealand adults with type 2 diabetes and poor Glycaemic control. J Diabetes Res.

[46] Yin J, Yeung R, Luk A, Tutino G, Zhang Y, Kong A, Chung H, Wong R, Ozaki R, Ma R, Tsang CC, Tong P, So W, Chan J (2016). Gender, diabetes education, and psychosocial factors are associated with persistent poor glycemic control in patients with type 2 diabetes in the joint Asia diabetes evaluation (JADE) program. J Diabetes.

[47] Lewis CP, Newell JN (2014). Patients' perspectives of care for type 2 diabetes in Bangladesh -a qualitative study. BMC Public Health.

[48] Kato A, Fujimaki Y, Fujimori S, Izumida Y, Suzuki R, Ueki K, Kadowaki T, Hashimoto H (2016). A qualitative study on the impact of internalized stigma on type 2 diabetes self-management. Patient Educ Couns.

[49] Halkoaho A, Kangasniemi M, Niinimki S, Anna MP (2014). Type 2 diabetes patients' perceptions about counselling elicited by interview: is it time for a more health-oriented approach?. Eur Diabetes Nurs.

[50] Liu C, Xu S, Ming J, Jia A, Wei Y, Li H, Jiao Y, Song M, Zhao Y, Du Y, Yang W, Lu X, Shi S, Tong H, Jia G, Zhao G, Wang L, Zhang M, Wang J, Liu W, Fang L, Dong F, Ji Q (2018). Differences between the perspectives of physicians and patients on the potential barriers to optimal diabetes control in China: a multicenter study. BMC Health Serv Res.

[51] de Silva D (2011). Helping people help themselves: a review of the evidence considering whether it is worthwhile to support self-management.

[52] Cradock KA, ÓLaighin G, Finucane FM, Gainforth HL, Quinlan LR, Ginis KA (2017). Behaviour change techniques targeting both diet and physical activity in type 2 diabetes: a systematic review and meta-analysis. Int J Behav Nutr Phys Act.

[53] Huang ES, Gorawara-Bhat R, Chin MH (2005). Self-reported goals of older patients with type 2 diabetes mellitus. J Am Geriatr Soc.

[54] Goetz K, Szecsenyi J, Campbell S, Rosemann T, Rueter G, Raum E, Brenner H, Miksch A (2012). The importance of social support for people with type 2 diabetes - a qualitative study with general practitioners, practice nurses and patients. Psychosoc Med.

[55] Carbone ET, Rosal MC, Torres MI, Goins KV, Bermudez OI (2007). Diabetes self-management: perspectives of Latino patients and their health care providers. Patient Educ Couns.

[56] Grant JS, Steadman LA (2016). Barriers to diabetes self-management among rural individuals in the workplace. Workplace Health Saf.

[57] Ruston A, Smith A, Fernando B (2013). Diabetes in the workplace - diabetic's perceptions and experiences of managing their disease at work: a qualitative study. BMC Public Health.

[58] Li X, Krumholz HM, Yip W, Cheng KK, De Maeseneer J, Meng Q, Mossialos E, Li C, Lu J, Su M, Zhang Q, Xu DR, Li L, Normand ST, Peto R, Li J, Wang Z, Yan H, Gao R, Chunharas S, Gao X, Guerra R, Ji H, Ke Y, Pan Z, Wu X, Xiao S, Xie X, Zhang Y, Zhu J, Zhu S, Hu S (2020). Quality of primary health care in China: challenges and recommendations. Lancet.

[59] Peng X, Guo X, Li H, Wang D, Liu C, Du Y (2022). A qualitative exploration of self-management behaviors and influencing factors in patients with type 2 diabetes. Front Endocrinol.

[60] Matthews SM, Peden AR, Rowles GD (2009). Patient-provider communication: understanding diabetes management among adult females. Patient Educ Couns.

[61] Al-Qazaz HK, Hassali MA, Shafie AA, Syed Sulaiman SA, Sundram S (2011). Perception and knowledge of patients with type 2 diabetes in Malaysia about their disease and medication: a qualitative study. Res Social Adm Pharm.

[62] Lai WA, Chie WC, Lew-Ting CY (2007). How diabetic patients' ideas of illness course affect non-adherent behaviour: a qualitative study. Br J Gen Pract.

[63] Powers MA, Bardsley JK, Cypress M, Funnell MM, Harms D, Hess-Fischl A, Hooks B, Isaacs D, Mandel ED, Maryniuk MD, Norton A, Rinker J, Siminerio LM, Uelmen S (2020). Diabetes self-management education and support in adults with type 2 diabetes: a consensus report of the American Diabetes Association, the Association of Diabetes Care & education specialists, the academy of nutrition and dietetics, the American Academy of family physicians, the American Academy of PAs, the American Association of Nurse Practitioners, and the American Pharmacists Association. Diabetes Educ.

[64] Young-Hyman D, de Groot M, Hill-Briggs F, Gonzalez JS, Hood K, Peyrot M (2016). Psychosocial Care for People with Diabetes: a position statement of the American Diabetes Association. Diabetes Care.

[65] Nicolucci A, Kovacs Burns K, Holt RI, Comaschi M, Hermanns N, Ishii H, Kokoszka A, Pouwer F, Skovlund SE, Stuckey H, Tarkun I, Vallis M, Wens J, Peyrot M, DAWN2 Study Group (2013). Diabetes attitudes, wishes and needs second study (DAWN2™): cross-national benchmarking of diabetes-related psychosocial outcomes for people with diabetes. Diabet Med.

[66] Berry E, Lockhart S, Davies M, Lindsay JR, Dempster M (2015). Diabetes distress: understanding the hidden struggles of living with diabetes and exploring intervention strategies. Postgrad Med J.

[67] Fisher L, Hessler D, Glasgow RE, Arean PA, Masharani U, Naranjo D, Strycker LA (2013). REDEEM: a pragmatic trial to reduce diabetes distress. Diabetes Care.

[68] Cheng L, Sit JW, Leung DY, Li X (2016). The association between self-management barriers and self-efficacy in Chinese patients with type 2 diabetes: the mediating role of appraisal. Worldviews Evid-Based Nurs.

