# Supplementary material for: Exploring facilitators and barriers to self-management engagement of Chinese people with type 2 diabetes mellitus and poor blood glucose control: a descriptive qualitative study
Source: BMC Endocr Disord. 2022 Nov 26;22:294. doi: 10.1186/s12902-022-01214-0 (PMC9701421; doi:10.1186/s12902-022-01214-0)
Supplement: Supplementary file 1 — Additional file 1. The guideline listed open-ended questions asked in the semi-structured interviews. [file 12902_2022_1214_MOESM1_ESM.docx]

Appendix.1 Interview guideline

| **Perceived susceptibility and severity** |
| --- |
| Could you please talk about your perceptions of your poor blood glucose control? |
| **Perceived benefits** |
| What do you think about lifestyle management? |
| What do you think about medication therapy? |
| What do you think about blood glucose monitoring? |
| **Perceived barriers** |
| How is the practice of your lifestyle management? |
| How is the engagement of your medication therapy? |
| How is the conduction of your blood glucose monitoring? |
